# Measuring of quality of care in patients with stroke and acute myocardial infarction: An application of algebra effectiveness model

**DOI:** 10.1097/MD.0000000000015353

**Published:** 2019-05-17

**Authors:** Kyoung Hee Cho, Chung Mo Nam, Sang Gyu Lee, Tae Hyun Kim, Seon-Heui Lee, Eun-Cheol Park

**Affiliations:** a Department of Public Health, Graduate School; b Institute of Health Services Research, College of Medicine; c Health Insurance Policy Research Insititue, National Health Insurance Service, Wonju; d Department of Biostatistics, College of Medicine; e Graduate School of Public Health, Yonsei University, Seoul; f Department of Nursing Science, College of Nursing, Gachon University, Incheon; g Department of Preventive Medicine, College of Medicine, Yonsei University, Seoul, Korea.

**Keywords:** acute myocardial infarction, algebra effectiveness model, in-hospital mortality, quality of care, stroke

## Abstract

Ischemic stroke, hemorrhagic stroke, and acute myocardial infarction (AMI) are diseases with golden hour. This study aimed to identify and compare factors that affect in-hospital mortality in patients with stroke and AMI who admitted via the emergency department.

This study used the Korean National Health Insurance claims data from 2002 to 2013. The study sample included 7693 patients who had an ischemic stroke, 2828 patients who had a hemorrhagic stroke, and 4916 patients with AMI who were admitted via the emergency departments of a superior general hospital and general hospital, did not transfer to another hospital or come from another hospital, and were aged ≥20 years. This study was analyzed by using Cox's proportional hazards frailty model.

Five hundred (6.5%) of 7693 patients with ischemic stroke, 569 (20.1%) of 2828 patients with hemorrhagic stroke, and 399 (8.1%) of 4916 patients with AMI were dead. The clinical factors were associated with in-hospital mortality such as age, CCI, hypertension, and diabetes of patient characteristics. In treatment characteristics, performing PCI and weekday admission was associated with in-hospital mortality (aHR, 0.43; 95% CI, 0.27–0.67; aHR, 1.42; 95% CI, 1.14–1.77, respectively). In hospital characteristics, the volume, the proportion of transferred patient to other hospital and ratio of beds per one nurse was associated with in-hospital mortality.

Clinical factors of patient characteristics, intervention such as performing PCI and reducing ICP of treatment characteristics, and the volume, transferred rate, and the number of nurse of hospital characteristics were associated with in-hospital mortality.

## Abstract 2

Supplemental Digital Content is available in the text

## 1 Introduction

The growing demand for health care, increasing treatment costs, constrained resources, and evidence of variations in clinical practice have increased the interest in measuring and improving the quality of health care in many countries.^[1]^ Quality of care can be defined in various ways depending on perspectives, and how quality is to be defined is an important issue. Until 1980s, basic approach for measuring of quality of care was to use a model of Donabedian in aspects of structure, process, and outcome.^[2]^ According to this theory, structure, process, and outcome can be indicator of quality of care. However, looking at history of quality assurance,^[3–8]^ approach for measuring of quality of care changed gradually that quality of care measured in terms of structure in early stage, and measured in aspect of process, and tendency have showed that measuring of quality of care emphasized on outcomes. Measuring structure and process is relative easy but there is a limitation in terms of indirect assessment. In contrast, measuring outcomes as quality of care has an advantage that can directly evaluate an effectiveness of quality of care. However, the three dimensions are intertwined, but their relative utility depends on context.^[9]^ Outcomes that are not linked to specific medical practices provide little guidance for developing quality-improvement strategies.^[10]^ Furthermore, comparing outcomes across groups frequently requires adjustment for patient risk and the recognition that some patients are sicker than others.^[11]^ For these reasons, Lisa Iezzoni provides a conceptual model of the summation of patient factors, treatment effects, and random events that produce health outcomes.^[11,12]^ This conceptual model is referred to as the “Algebra Effectiveness.”^[12]^ This model is based on the viewpoint that in-hospital patient outcome is caused by the sum of three components. To assess hospital net quality, factors such as patient factors are removed that could affect health outcome.

In Korea, to improve quality of care for stroke and acute myocardial infarction (AMI), the Health Insurance Review and Assessment Service (HIRA) demonstrated pay for performance (P4P) from July 2007 to December 2010 for AMI and stroke for superior general hospitals. Despite several advances in AMI and stroke care over the last decade, Cardiovascular and cerebrovascular diseases accounts for a quarter of the total mortality. These diseases were the second and third leading causes of mortality, being responsible for 52.4 and 48.2 deaths per 1,000,000 people in Korea, respectively.^[13]^ Especially stroke is the first leading cause of mortality as single disease in Korea,^[1]^ and is the second leading cause of death worldwide.^[14]^

The aim of this study was to identify and compare patient, treatment, and hospital characteristics that affect the in-hospital mortality of patients with who were admitted via the emergency department due to stroke and AMI by an application of algebra effectiveness model.

## 2 Methods

### 2.1 Data source

The dataset was obtained from the Korean National Health Insurance (KNHI) claims database from 2002 to 2013. The National Health Insurance Corporation collects cohort data representative of the country's population.^[15]^ These data include information on 10,250,340 patients. These subjects represent a stratified random sample selected according to age, sex, region, health insurance type, income quintile, and individual total medical costs based on the year 2002. The database includes information on reimbursement for each medical service, including basic patient information, an identifier for the clinic or hospital, a disease code, costs incurred, results of health screening, personal/family history, health behaviors, and information related to death. These data are publicly available for research purposes. Ethical approval for this study was granted by the institutional review board of the Graduate School of Public Health, Yonsei University (2-1040939-AB-N-01-2016-402).

### 2.2 Study sample

The total number of individuals admitted to acute care hospitals, including superior general hospitals and general hospitals, via the emergency department without dropping by other health-care institutions due to ischemic stroke, hemorrhagic stroke, and AMI. The Korean health-care delivery system is classified into 3 steps based on fee-for-services as the reimbursement mechanism as follows: clinics function as primary care institutions, hospitals function as secondary care institutions, and general hospitals function as tertiary care institutions. Our study sample was included only patients who utilized general hospitals, 20 years old or more, and was not transferred to other hospitals. Because clinics were likely to receive only low-risk or a limited number of patients, and in case of transferring patients, each hospital's net quality or performance cannot be measured, and time to treatment was delayed. To determine real stroke and AMI patients, patients with stroke and AMI as a principal or secondary diagnosis were identified by searching for codes of the *International Classification of Diseases, 10th Revision* (*ICD-10*); medication information; and clinical test information. According to the assessment report for AMI and stroke quality, in cases of ischemic stroke and AMI, the administration rates of anticoagulants and antiplatelet agents within 48 h and aspirin within 24 h are both almost close to 100%. In the case of AMI, almost all patients with suspected AMI received tests for cardiac enzymes such as creatine kinase MB fraction (CK-MB), troponins T and I, and myoglobin. In the case of hemorrhagic stroke, approximately 90% of these patients received intravenous antihypertensive drugs according to the guideline for hemorrhagic stroke. Otherwise, patients received mannitol to control their conditions or craniotomy to decrease intracranial pressure as intervention. The final study sample with ischemic stroke included 7693 participants, the final study sample with hemorrhagic stroke included 2828 participants, and the final study sample with AMI included 4916 participants (Supplemental Figure 1 to Figure 3).

### 2.3 Variables

#### 2.3.1 Dependent variables

The dependent variable in the present study was mortality upon in-hospital admission, and at 7 and 30 days after admission. Death was assumed to be the outcome of interest. Death was determined by linking inpatient records with death certificate records from the national death registry. The death certificate records indicated only the month and year of death; we had to determine whether the patient was dead at discharge. We defined in-hospital, 7-day, and 30-day mortalities as follows: First, we matched the discharge and death dates. If the discharge data month/year was the same as the death date, we determined if the patients acquired discharge medication or used any medical services after the discharge date. If they did not, we included them as cases as mortalities.

#### 2.3.2 Independent variables

Patient, treatment, and hospital characteristics were classified as covariates. Patient characteristics included age; sex; health insurance type (national health insurance or medical aid); income level; residential area (metropolitan/urban/rural); Charlson comorbidity index (CCI ≤ 1, 2, 3, or ≥4); arrival route (by emergency team/others); disability status (none/mild/severe); presence or absence of hypertension/hypertensive complications (with congestive heart failure and renal failure), diabetic complications (with coma lactic acidosis, renal complications, diabetic nephropathy, end-stage renal disease, ophthalmic complications, retinopathy, diabetic cataract, diabetic neuropathy, diabetic angiopathy with/without gangrene, with musculoskeletal and connective tissue complication, with periodontal complication, and hypoglycemia) and hyperlipidemia; and admission on a weekend or weekday. Treatment characteristics included administration of an intervention such as embolization, balloon, or stent (yes/no); administration of intravenous thrombolytic agents in case of ischemic diseases; use of intensive care unit service (yes/no); and administration of surgical procedures (yes/no) in case of hemorrhagic stroke. Hospital characteristics included ownership (public/educational/private), total number of patients admitted via the emergency department because of a corresponding condition per year (quintiles 1–5), proportion of transferred patients to another hospital (<5/5–9/10–14/15–19/≥20), number of beds (quintiles 1–5), patient-to-physician ratio (2.5:1/ 3.5:1/ 5.5:1/ 8.5:1/ >8.5:1), patient-to-nurse ratio (2.0:1/ 2.5:1/ 3.0:1/ 3.5:1/ 4.0:1/ 4.5:1/ >6.0:1), and hospital function (superior general hospital/general hospital). Only the comorbidity component of the CCI was calculated from entry of cohort to before occurrence of interested conditions. In addition, when we calculated CCI, we extracted diabetes scores. The weekend effect was investigated by determining whether patients were admitted via the emergency department on a Saturday or Sunday. In addition, patients who were admitted on an official national holiday were regarded as weekend admissions. The definition of variables shows the variables that used in this study (Supplemental Table 1).

### 2.4 Statistical analysis

Descriptive statistics were computed for all variables as follows: Frequencies and percentages of categorical variables were determined by using the chi-square test. The cumulative incidence for each dependent was estimated by using the Kaplan-Meier product limit method with log-rank tests. To investigate the association between patient/treatment/hospital characteristics and mortality, we performed a survival analysis by using Cox's proportional hazards frailty model, which included random effects to account for covariate hierarchy. This approach used a random effect to test for a hospital effect. This random effect can be thought of as a “frailty,” which increases a hospital's susceptibility to a short survival time when it is large and decreases this susceptibility when it is small. We determined the mortality variance and *P*-values among the hospitals. The variance and *P* values were 0.126 and 0.050 for ischemic stroke, 0.081 and 0.04 for AMI, and 0.109 and 0.021 for hemorrhagic stroke, respectively.

The equation λ (*t*|**x**) = *z*λ0 (*t*)exp (**x**β) describes the frailty model, where **x** is the covariate matrix, β is the fixed effect vector, and *z* is a random variable representing an unknown random effect related to each hospital, with unit mean and variance ξ. These random effects act multiplicatively on the baseline hazard, and large ξ values reflect a great degree of heterogeneity among hospitals. For model distribution purposes, we assumed that the frailties were distributed according to a gamma distribution. One attractive feature of the gamma distribution is that it is mathematically tractable. All the statistical analyses were performed by using SAS 9.4 (SAS institute, Inc., Cary, NC, USA).

## 3 Results

Table 1 shows how the three diseases differ in distribution and the general characteristics used in this study. The proportion of individuals who had a hemorrhagic stroke (≥70) was lower than that of patients who had an ischemic stroke and AMI (30.2%, 49.0%, and 41.4%, respectively). Regarding sex, the proportion of males was highest among those with AMI, followed by ischemic and hemorrhagic stroke (63.0%, 54.0%, and 49.6%, respectively). In in-hospital mortality, 500 patients (6.5%) died from ischemic stroke; 399 (8.1%), from AMI; and 569 (20.1%), from hemorrhagic stroke (Supplemental Table 2).

**Table 1:**
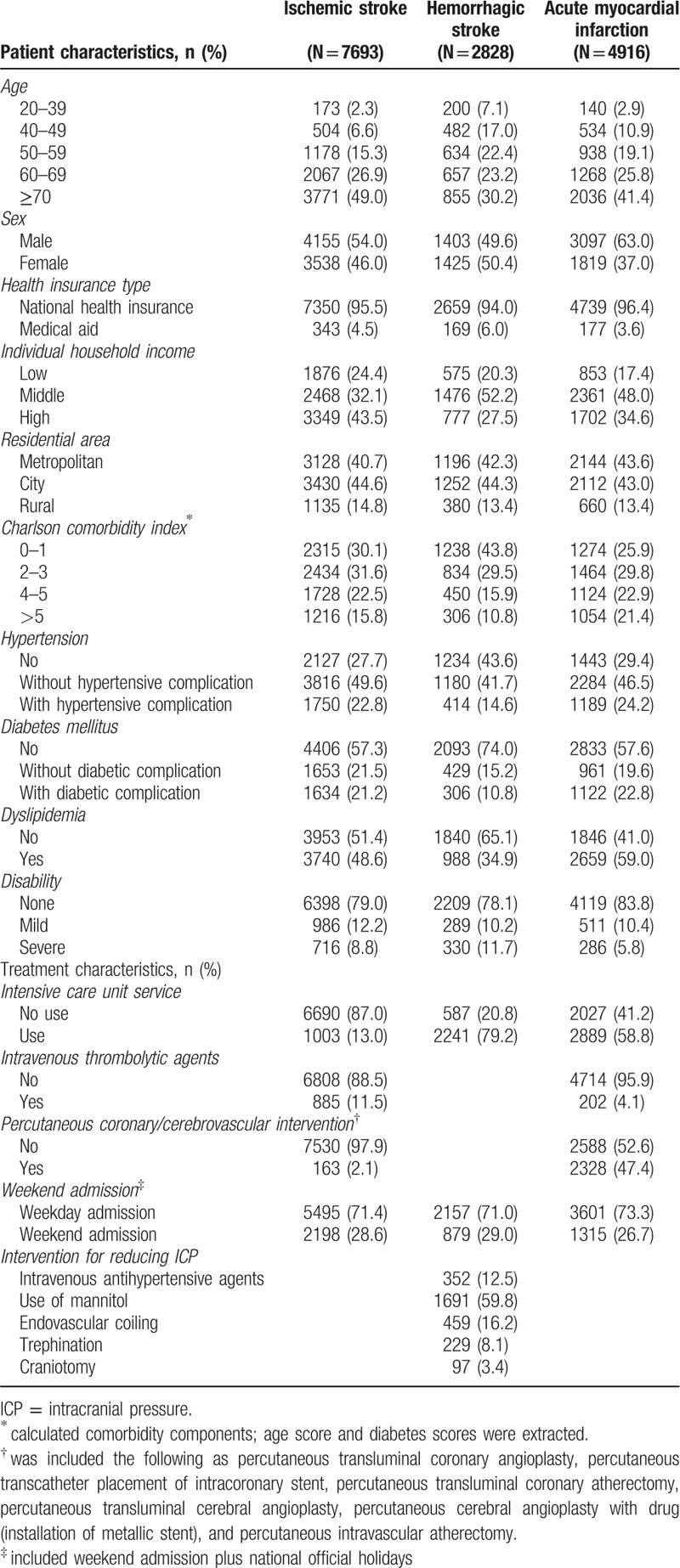
Patient characteristics who admitted via emergency department, stratified by disease.

**Figure 1:**
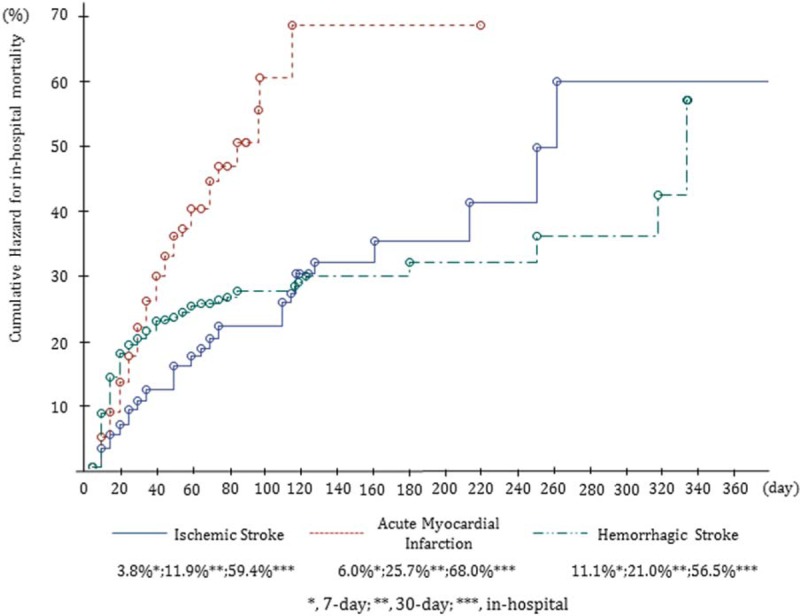
The cumulative hazards, stratified to diseases.

Figure 1 shows the cumulative hazards stratified according to disease. The in-hospital cumulative hazards were 59.4% for ischemic stroke, 68.0% for AMI, and 56.5% for hemorrhagic stroke. The in-hospital cumulative mortality was highest for AMI.

Table 2 presents the adjusted hazard ratios of in-hospital mortality from Cox's proportional frailty hazard model for predicting patient characteristics and in-hospital mortality in ischemic stroke, hemorrhagic stroke, and AMI. The HRs of the patients with low and middle incomes were 1.30 (95% CI, 1.02–1.65) and 1.33 (95% CI, 1.08–1.64), respectively. Regarding sex, the HRs of the males were 0.82 for ischemic stroke (95% CI, 0.68–0.99) and 1.25 (95% CI, 1.06–1.48) for hemorrhagic stroke compared with the females. The risk of in-hospital mortality showed contrary result in relation to sex. The HR of any age group was lower than that of the reference group (≥70 years old) in AMI. Table 3 shows the association between treatment characteristics and in-hospital mortality. Regarding the use of intensive care unit service, use of intensive care unit service was associated with in-hospital mortality in all three diseases. In ischemic stroke, the HR of use of the intensive care unit service was 5.54 (95% CI, 4.61–6.65) for in-hospital mortality. Trephination and craniotomy were associated with increasing risks of in-hospital mortality. Weekend admission was associated with mortality only in AMI. The adjusted HR of weekend admission was 1.42 (95% CI, 1.14–1.77) for in-hospital mortality. In addition, performing PCI was associated with in-hospital mortality in AMI. The HR of performing PCI was 0.42 (95% CI, 0.33–0.54) for in-hospital mortality. Table 4 shows the association between hospital characteristics and in-hospital mortality. The characteristic of the funding source, hospital volume, and patient-to-nurse ratio were associated with in-hospital mortality. In all of diseases, hospital volume was associated with in-hospital mortality. Compared with the reference hospital with a volume within quintile 5, the adjusted HRs of the hospitals with volumes within quintiles 1 and 2 were 2.69 (95% CI, 1.17–3.81) and 1.49 (95% CI, 1.13–1.96) in hemorrhagic stroke and the adjusted HRs of the hospitals with volumes within quintiles 1 and 2 were 1.85 (95% CI, 1.21–2.83) and 1.98 (95% CI, 1.35–2.90) in ischemic stroke, respectively. The adjusted HR of hospitals that the proportion of transferred patient to other hospital was 20% or more was 1.31 (95% CI, 1.00–1.74) in hemorrhagic stroke, comparing to the reference group that the proportion of transferred patient to other hospital was 5% or less. Regarding the patient-to-nurse ratio, the risk of in-hospital mortality decreased as number of nurses increased in ischemic stroke. Compared with the reference hospital, where the patient-to-nurse ratio was 2.0:1, the HRs for 2.5:1, 3.0:1, 3.5:1, 4.01:1, 4.5:1, and >6.0:1 were 2.48 (95% CI, 0.90–6.84), 3.26 (95% CI, 1.21–8.81), 3.57 (95% CI, 1.30–9.84), 3.77 (95% CI, 1.33–10.72), 3.09 (95% CI, 1.05–9.12), and 4.36 (95% CI, 1.50–12.66), respectively.

**Table 2:**
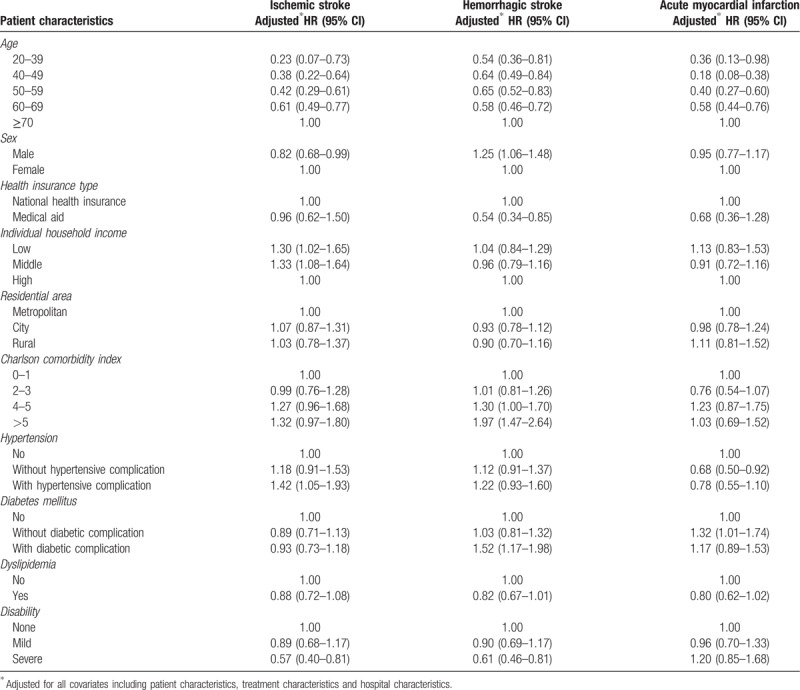
Association between patient characteristics and in-hospital mortality, stratified by diseases.

**Table 3:**
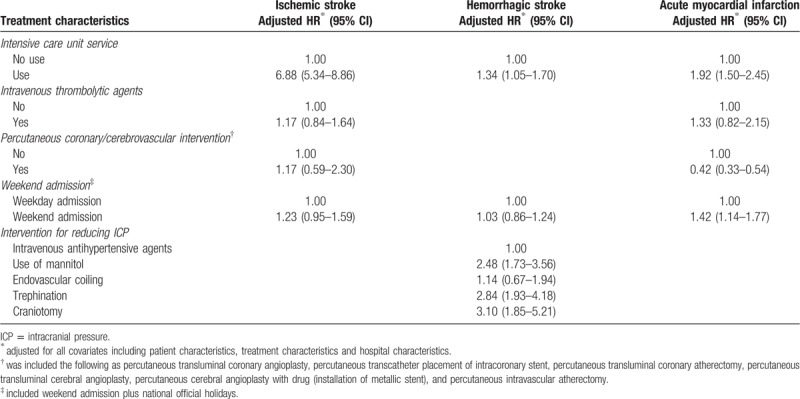
Association between treatment characteristics and in-hospital mortality, stratified by diseases.

**Table 4:**
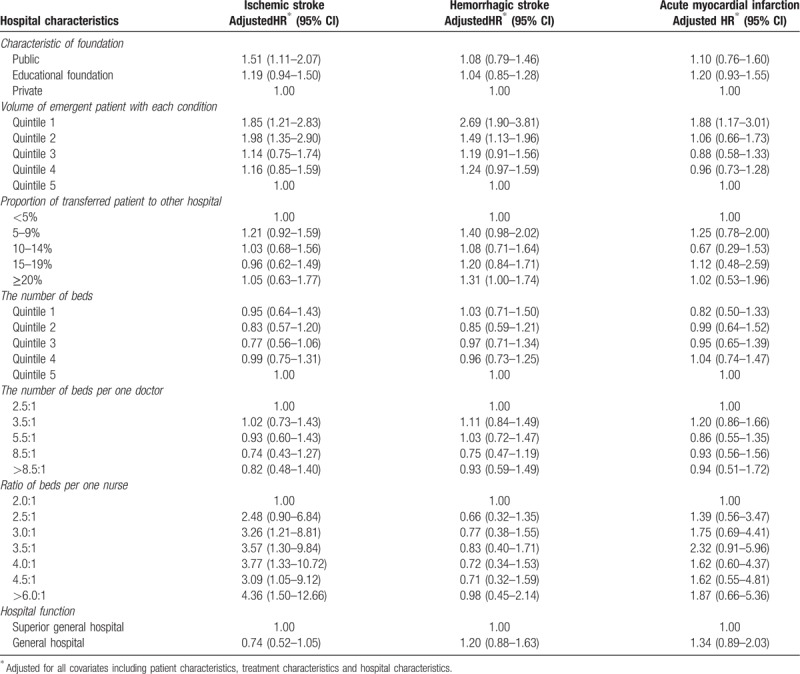
Association hospital characteristics and in-hospital mortality, stratified by diseases.

## 4 Discussion

This study examined the associations of patient, treatment, and hospital characteristics with in-hospital mortality by using representative nationwide cohort data. In terms of patient characteristics, age, individual household income, and with hypertensive complication were associated with in-hospital mortality in ischemic stroke, age, sex, health insurance type, CCI, and with diabetic complication were associated with mortality in hemorrhagic stroke, and age, and diabetes without diabetic complication were associated with mortality in AMI. All of the three conditions, the risk of in-hospital mortality was higher for the patients who used ICU. In AMI, the HR for in-hospital mortality was higher for the patients admitted on a weekend than for those admitted on a weekday. In terms of hospital characteristics, all of the three conditions, the volume of emergent patient with each condition was associated with in-hospital mortality, and in hemorrhagic stroke, the rate of transferred patient to other hospital was associated with mortality. In ischemic stroke, the risk of mortality was decreased as the patient-to-nurse ratio increased in hemorrhagic stroke.

In the analysis of the association between patient characteristics and mortality, diabetes and dyslipidemia were not statistically significant factors of stroke. However, the risk of mortality was higher for the patients with than for those without hypertension. Previous studies found that the population-attributable risk (PAR) of hypertension was 22.7% to 28.5%, but the PAR of diabetes was 14.6%.^[16]^ The difference was >10%. Our results were consistent with those of previous studies that showed that the PAR of hypertension was higher than the PAR of diabetes in stroke. The PAR of diabetes was 5%^[17]^ and that of hypertension was 25% for AMI. The PAR of hypertension was higher than that of diabetes. These results were consistent with the results of INTERHEART study^[18]^ and INTERSTROKE study.^[19]^ The both previous studies showed that the modifiable risk factors accounted for 90% or more in stroke and AMI, and the PAR of hypertension was higher than that of diabetes. Our results showed that the risk of mortality was lower for the patients with than for those without hypertension, and the risk of mortality in AMI was higher for the patients with than for those without diabetes. AMI is a more common disease than stoke and occurs easily in young people. In Korea, according to the report of the Korean National Nutrition and Health Examination, the perception and treatment rates of hypertension or diabetes are low for young people.^[20]^ Therefore, patients without hypertension or diabetes might include patients who do not recognize their diseases. We could not consider the dynamic interaction of all three conditions according to medication compliance.

The HR of mortality was higher for the patients admitted to the intensive care unit than for those who were not in all three diseases. Admission in the intensive care unit might proxy for disease severity itself. However, the risk of mortality among the patients admitted to the intensive care unit was highest in ischemic stroke, followed by AMI, and the lowest in hemorrhagic stroke. The difference in admission rate between the patients admitted to the intensive care unit and those who were not was the greatest in ischemic stroke, followed by AMI and then hemorrhagic stroke. We thought the difference likely reflects the difference in disease severity between the two patient groups. Performing percutaneous coronary intervention (PCI) reduced the risk of mortality in AMI.^[21]^ PCI was one of the most important processes. Moreover, considering that the cumulative mortality was the greatest of all three conditions, performing PCI was the most effective way of reducing the risk of mortality. For the patients admitted to a superior general hospital because of ischemic stroke and AMI, we could observe the weekend effect.^[22–24]^ Especially in the case of AMI, if we consider that performing PCI could decrease the risk of mortality, the availability of personal resources that can perform a PCI during a weekend or holiday might be a critical factor.

In case of ischemic stroke, the risk of mortality was higher for the patients admitted to hospitals with public sources of funding or to hospitals with sources of funding for educational purposes than for those admitted to hospitals with private funding. The hospitals with public funding tended to be weaker financially than the other hospitals, which leads to poor quality of care.^[25]^ We observed a steady increase in cumulative mortality regardless of an acute or chronic phase. Our findings suggest that the overall hospital quality was more important during the whole admission period relative to the other factors of ischemic stroke. Hospitals with private funding are likely to invest in improving quality of care. Therefore, considering the characteristic of ischemic stroke, we thought that the characteristic of funding sources is associated with mortality. The patient-to-nurse ratio was associated with mortality in ischemic stroke. We thought this variable was likely to represent the overall quality of care provided by hospitals. AMI and hemorrhagic stroke are diseases that need specialized techniques such as PCI or craniotomy. In these two diseases, hospital volume and transfer rate to another hospital were associated with mortality. This finding was consistent with those of previous studies.^[26–29]^ In addition, in the case of AMI, the patient-to-physician ratio was associated with mortality for some groups. Considering these results, we think organizational or individual competence might be a critical factor. In hemorrhagic stroke, the patient-to-nurse or physician ratio was greater, but the hazard ratio was smaller. This finding contradicts the general idea. Thus, this study performed two tests to resolve the issues that led to these results.

Some issues could be raised related with the study methods. First limitation was an issue related to data source. These data were extracted from a national health insurance claims database from 2002 to 2013. These subjects represent a stratified random sample selected according to age, sex, region, health insurance type, income quintile, and individual total medical cost based on the year 2002. These data were not from a representative organization. Therefore, the definitions of the hospital characteristics could not be generalized. Second was the selection of the study sample. The study data were obtained from a claims database. To determine the study sample, we used ICD-10 code as primary diagnostic criteria. The diagnostic accuracy in the KNHI claims data is roughly 70%.^[30]^ Although this study tried to identify throughout real patients by using medication and clinical test information, the diagnostic accuracy might have been compromised in the study. In addition, we could not consider the time of first medical contact, the time to electrocardiogram (ECG) and PCI or thrombolysis, because this information can be obtained from medical records. Third was to define dependent variable. This study determined the death date by examining whether the patient received discharge medication and/or used medical services after discharge. Although the death date month/year and the discharge date month/year were identical, it is possible that some patients were discharged against medical advice, did not take discharge medication, and did not use medical services after discharge. Finally, in stroke and AMI, pre-hospital intervention is one of the most important factors. However, this study could not consider pre-hospital intervention because this study used claims data.

Despite these limitations, this study used a representative data and included all data on all-cause mortality. This study used socioeconomic status, which could affect mortality, unlike studies that used administrative data. In addition, this study used a robust study design and Cox's proportional hazard frailty model to investigate factors at the individual, treatment, and hospital levels that could impact mortality.

In conclusions, hypertension, diabetes, and dyslipidemia were associated with increased risk of mortality in stroke and AMI. These factors are preventable; thus, policies should focus on primary prevention by changing to healthy lifestyles. In AMI, performing PCI was strongly associated with reduced risk of mortality. In hospital factors, the ratio of number of patient per one nurse was associated with mortality in ischemic stroke. The volume was associated with mortality in all three diseases, and the transferred rate was associated with mortality in hemorrhagic stroke. To secure experts, we need policies that can supply experts and train the experts at national level who can perform intervention such as PCI. In case of diseases that organizational and hospital staff's competence is important, an introduction of system is needed that can share the list of organization which can manage patients with these diseases between institution.

## Acknowledgments

This study was based on Kyoung Hee Cho's doctoral dissertation.

## Author contributions

**Conceptualization:** Kyoung Hee Cho.

**Formal analysis:** Kyoung Hee Cho, Chung Mo Nam.

**Investigation:** Kyoung Hee Cho.

**Methodology:** Kyoung Hee Cho, Chung Mo Nam.

**Project administration:** Eun-Cheol Park.

**Supervision:** Eun-Cheol Park.

**Validation:** Chung Mo Nam.

**Writing – original draft:** Kyoung Hee Cho.

**Writing – review & editing:** Sang Gyu Lee, Tae Hyun Kim, Seon-Heui Lee, Eun-Cheol Park.

## Supplementary Material

Supplemental Digital Content
