# Supplementary material for: Measuring of quality of care in patients with stroke and acute myocardial infarction: An application of algebra effectiveness model
Source: Medicine (Baltimore). 2019 May 17;98(20):e15353. doi: 10.1097/MD.0000000000015353 (PMC6531128; doi:10.1097/MD.0000000000015353)
Supplement: Supplemental Digital Content [file medi-98-e15353-s001.pptx]

## Slide 1
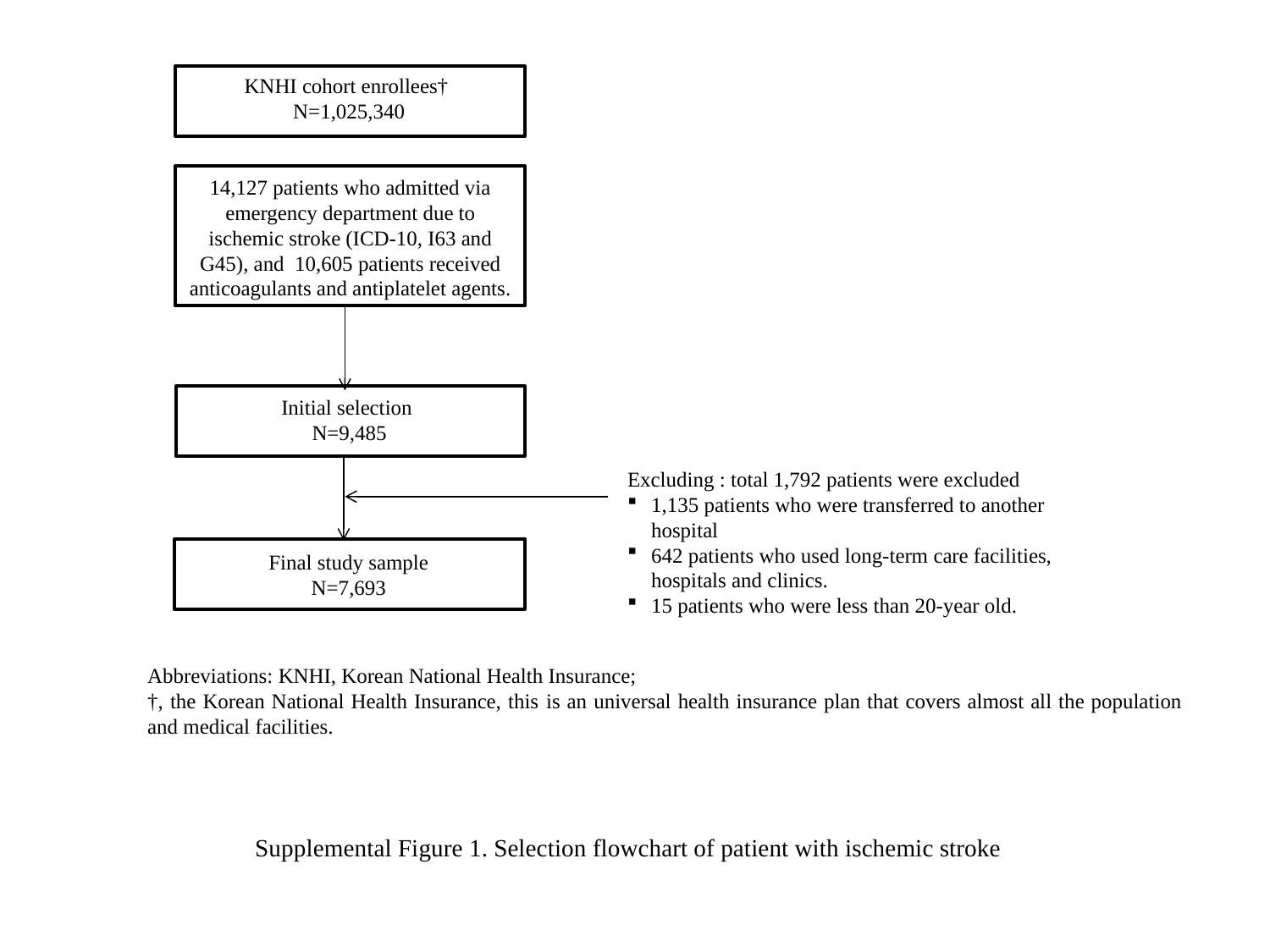

KNHI cohort enrollees†
N=1,025,340
14,127 patients who admitted via emergency department due to ischemic stroke (ICD-10, I63 and G45), and 10,605 patients received anticoagulants and antiplatelet agents.
Initial selection
N=9,485
Excluding : total 1,792 patients were excluded
1,135 patients who were transferred to another hospital
642 patients who used long-term care facilities, hospitals and clinics.
15 patients who were less than 20-year old.
Final study sample
N=7,693
Abbreviations: KNHI, Korean National Health Insurance;
†, the Korean National Health Insurance, this is an universal health insurance plan that covers almost all the population and medical facilities.
 Supplemental Figure 1. Selection flowchart of patient with ischemic stroke

## Slide 2
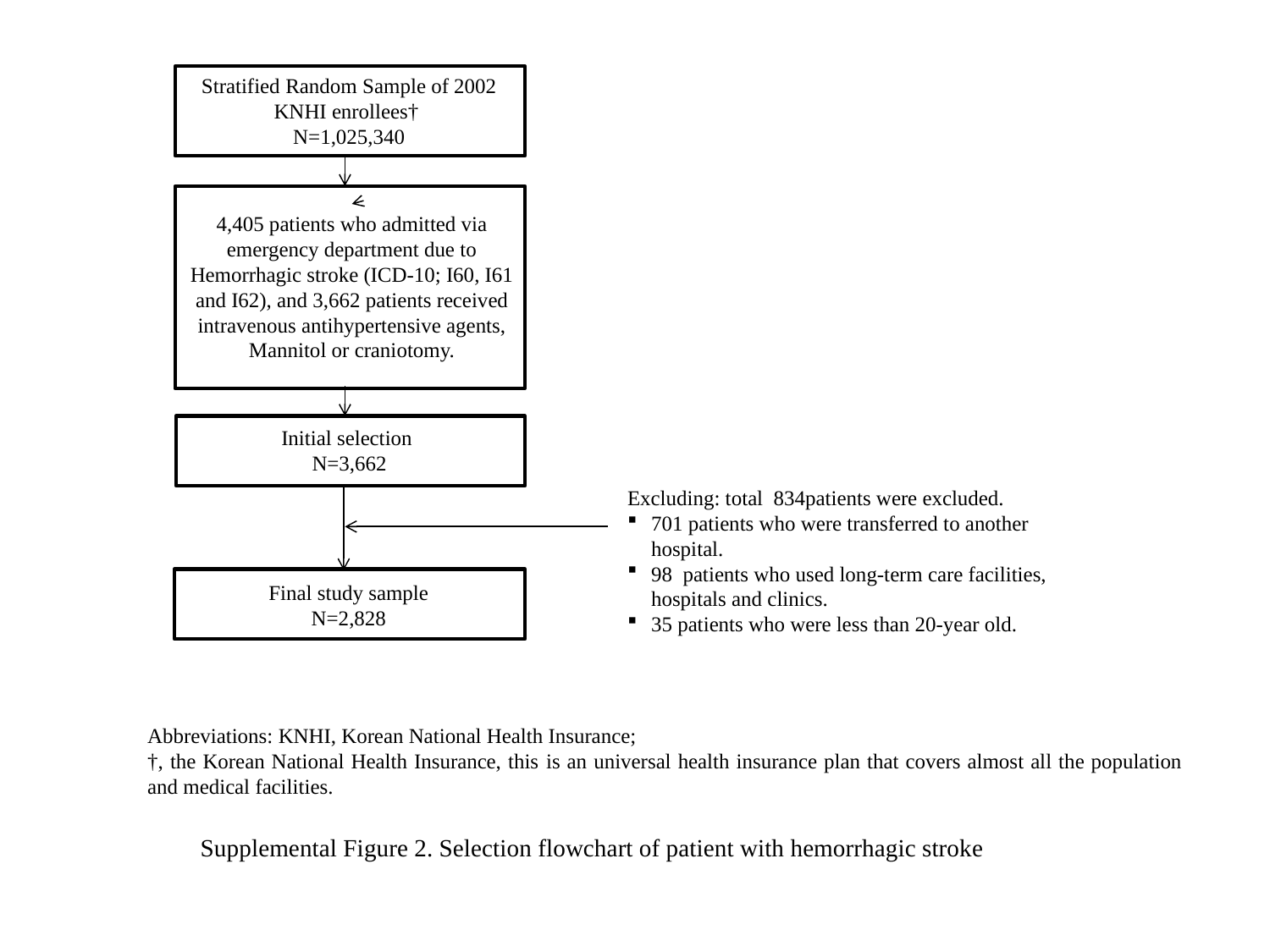

Stratified Random Sample of 2002 KNHI enrollees†
N=1,025,340
4,405 patients who admitted via emergency department due to Hemorrhagic stroke (ICD-10; I60, I61 and I62), and 3,662 patients received intravenous antihypertensive agents, Mannitol or craniotomy.
Initial selection
N=3,662
Excluding: total 834patients were excluded.
701 patients who were transferred to another hospital.
98 patients who used long-term care facilities, hospitals and clinics.
35 patients who were less than 20-year old.
Final study sample
N=2,828
Abbreviations: KNHI, Korean National Health Insurance;
†, the Korean National Health Insurance, this is an universal health insurance plan that covers almost all the population and medical facilities.
Supplemental Figure 2. Selection flowchart of patient with hemorrhagic stroke

## Slide 3
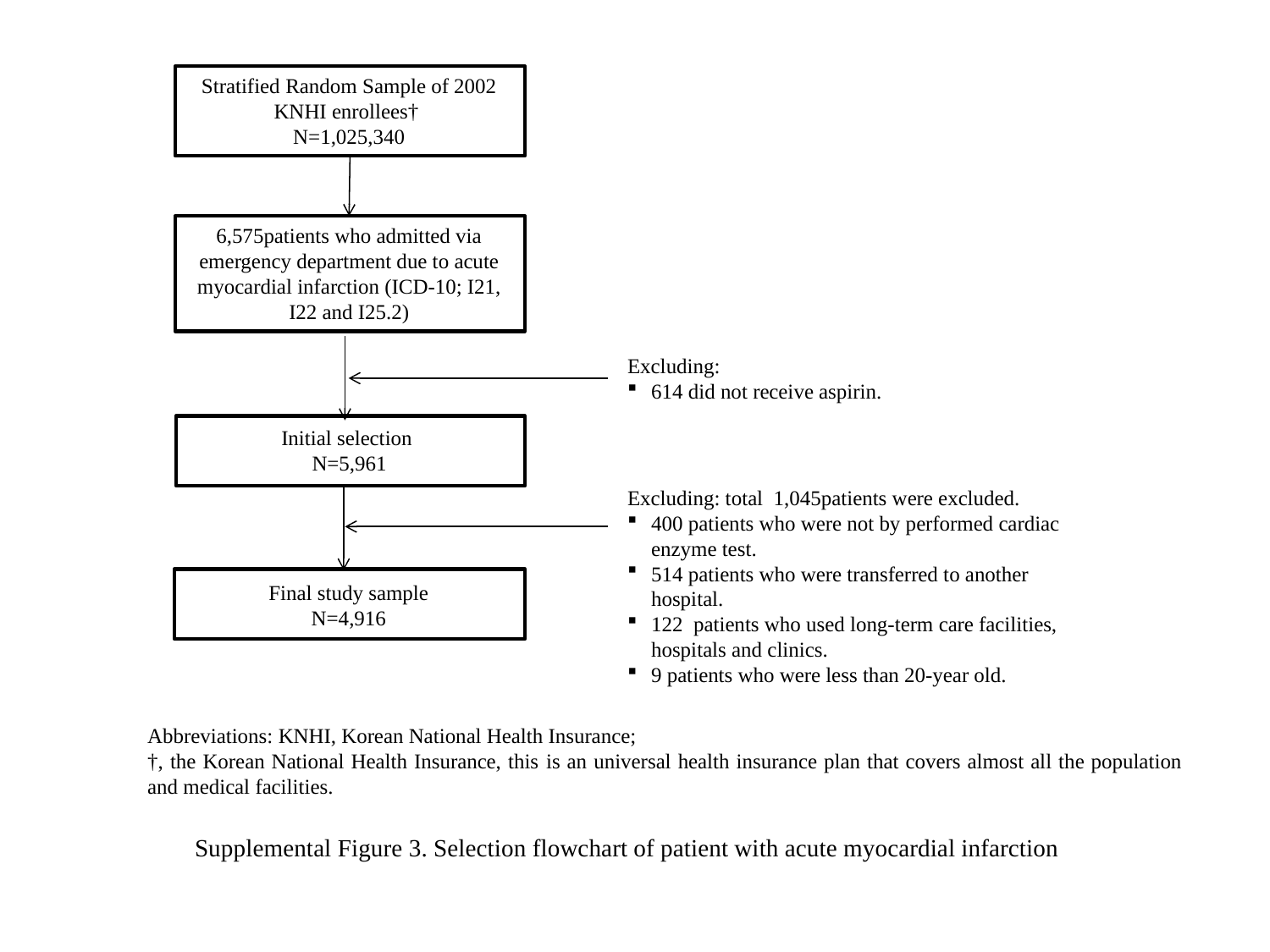

Stratified Random Sample of 2002 KNHI enrollees†
N=1,025,340
6,575patients who admitted via emergency department due to acute myocardial infarction (ICD-10; I21, I22 and I25.2)
Excluding:
614 did not receive aspirin.
Initial selection
N=5,961
Excluding: total 1,045patients were excluded.
400 patients who were not by performed cardiac enzyme test.
514 patients who were transferred to another hospital.
122 patients who used long-term care facilities, hospitals and clinics.
9 patients who were less than 20-year old.
Final study sample
N=4,916
Abbreviations: KNHI, Korean National Health Insurance;
†, the Korean National Health Insurance, this is an universal health insurance plan that covers almost all the population and medical facilities.
Supplemental Figure 3. Selection flowchart of patient with acute myocardial infarction
